# Case Report of Toxic Shock-like Syndrome Associated with Mixed *Staphylococcus aureus*, *Streptococcus halichoeri* and *Dermatophilus* spp. Infection in a Dog

**DOI:** 10.3390/vetsci12080764

**Published:** 2025-08-16

**Authors:** Carmen Negoiță, Veronica Ciupescu, Laurențiu Mihai Ciupescu, Valentina Negoiță

**Affiliations:** 1 Faculty of Veterinary Medicine, University of Agronomic Sciences and Veterinary Medicine, Bucharest 105 Independence Splai, District 5, 050097 Bucharest, Romania; carmen.negoita@fmvb.usamv.ro; 2 Institute for Hygiene and Veterinary Public Health, Sos. Oltenitei 35-37, 041293 Bucharest, Romania; ciupescu_veronica@yahoo.com; 3 Institute of Oncology “Prof. Dr. Alexandru Trestioreanu”, 252 Fundeni Road, District 2, 022328 Bucharest, Romania; negoitavalentina75@ymail.com

**Keywords:** antibiotic therapy, *Dermatophilus*, PCR, *Streptococcus halichoeri*, TSS, *Staphylococcus aureus*

## Abstract

Toxic shock syndrome (TSS) is a serious, often fatal disease, rarely occurring in dogs via infection with *Staphylococcus* and *Streptococcus*. The development of TSS is mainly dependent on the presence of bacterial toxins recognized to be potent superantigens causing the release of massive amounts of host inflammatory cytokines, notably TNF-α, progressing to high fever, hypotension, haemoconcentration, thrombosis and neutrophil and endothelial activation with multiple organ failure. Rarely, TSS is associated with erythematous and exfoliative dermatitis progressing to ulceration with extremely extensive dermo-epidermal detachment, which is often very painful. Like in humans, very little is known about the transmission and prevention of this condition. In our paper, a case of TSS-like caused by a mixed bacterial infection with *Staphylococcus aureus*, *Streptococcus halichoeri* and *Dermatophilus* spp. has been described in an 11 year-old, cross-breed male dog, most probably following injury due to biting and fighting. Lesions consisted of severe and diffuse ulceration on the dorsum, and bacterial culture/cytology led to the isolation and identification of Gram-positive staphylococci and streptococci associated with an intense neutrophil reaction. *Dermatophilus* spp. was presumed morphologically based on cytological preps, not by culture or molecular analysis. PCR demonstrated the presence of the *nuc* thermonucleaze gene (for *S. aureus* confirmation) together with the genes encoding enterotoxin H (*seh*), protein A (*spa*), toxic shock syndrome toxin TSST-1 (*tst*) and methicillin resistance (*mec*C); the exfoliative toxins (*eta*, *etb*) were detected. Clinical signs, cytology, bacterial culture and the response to systemic antibiotic therapy were compatible with a TSS-like diagnosis. The patient has completely recovered after 1 year of treatment.

## Simple Summary

Toxic Shock Syndrome (TSS) is a threatening disease rarely occurring in human and animals, mainly caused by microbial toxins, such as lipopolysaccharide of Gram-negative bacteria and peptidoglycan and exotoxins of Gram-positive bacteria. Bacterial toxins are recognized as potent superantigens causing the release of massive amounts of host inflammatory mediators responsible for the clinical and pathologic features of septic shock (high fever, hypotension, thrombosis, immune activation, multiple organ failure). In this paper, we describe a case of TSS-like caused by mixed bacterial infection predominantly involving *Staphylococcus aureus*, in an old, male dog, following a biting injury. The most surprising clinical signs were dermatological lesions, which consisted of severe, diffuse and symmetrical skin ulceration on the dorsum concurrent with fever and depression. Routine laboratory and molecular techniques were helpful in the diagnosis. The treatment was challenging because of recurrent infections, but multiple, long-term antibiotic therapies finally led to complete recovery of the patient. Very little is known about the transmission and prevention of this condition. However, the zoonotic potential of the incriminated bacteria, especially *Staphylococcus aureus*, which is known to be the most versatile colonizer and multiple-host pathogen able to readily cross species barriers and adapt to new hosts, should not be excluded.

## 1. Introduction

Staphylococci are Gram-positive cocci recognized as significant commensal and opportunistic pathogens, commonly responsible for canine pyoderma, especially *S. pseudintermedius*. *S. aureus* does not typically colonize dog skin, but occasionally can lead to severe infections and more rarely toxic shock syndrome (TSS) due to sophisticated attack and defence mechanisms [1,2,3,4,5,6,7,8,9]. Surprisingly, *S. aureus* strains isolated from pets are mainly of human origin and passed between human owners and their animals [1,3,8]. New data highlighted *S. aureus* as the most versatile colonizer and multiple-host pathogen able to readily cross species barriers and adapt to new hosts and new niches through the acquisition of and/or loss mutations in mobile genetic elements (MGEs) [1,3,9]. *S. aureus* pathogenicity is based on a combination of toxin-mediated cytotoxicity, invasiveness, immune evasion and antibiotic resistance in correlation with host specific reactivity [2,3,5,6,7,9]. The development of staphylococcal TSS is mainly dependent on the presence of bacterial toxins recognized to be potent superantigens causing the release of massive amounts of host inflammatory cytokines, notably TNF-α. Both *S. aureus* and *S. pseudintermedius* are known to produce a number of superantigens, including staphylococcal enterotoxins (SEs), staphylococcal exfoliative toxin (ET) and toxic shock syndrome toxin-1 (TSST-1) [3,5,10]. These molecules are responsible for the clinical and pathologic features of septic shock, including high fever, hypotension, haemoconcentration, thrombosis, neutrophil and endothelial activation, non-specific T-cell proliferation and multiple organ failure, but also severe exfoliative dermatitis progressing to ulceration with extremely extensive dermo-epidermal detachment, which is often very painful [5,11,12,13,14].

*S. halichoeri* appears to be a member of the skin microbiota in dogs. It is commonly associated with skin infections, but rarely isolated as a monoculture, so its clinical significance remains uncertain. However, the pathogenic potential of *S. halichoeri* should not be neglected, being reported in severe postoperative infections, abscesses, sinusitis, otitis media and necrotic pyoderma in humans, dogs and other animal species [2,15]. Surprisingly, *S. halichoeri* isolates from dog and human were found to be genetically similar indicating the zoonotic potential of this bacterium [15,16,17].

By contrast, *Dermatophilus* spp. is an actinomycete pathogen not found as a normal skin inhabitant in dogs, and it can be transmitted through contact with another carrier animal or via acquisition from the soil. Generally, chronic trauma associated with insect bites, moisture and debilitating diseases may predispose to an individual to dermatophilosis, characterized by crusting, erythematous and suppurative and ulcerative dermatitis in more severe forms. These skin lesions are caused by bacterial phospholipases and extracellular proteases, with a role in epidermal penetration, intense neutrophil infiltration and possibly inactivation of host immune response [18].

In this paper, a case of TSS-like caused by mixed infection with aforementioned bacterial species has been described in an 11 year-old, cross-breed intact male dog, most probably following injury to the dog due to biting and fighting.

## 2. Materials and Method

**Figure 1:**
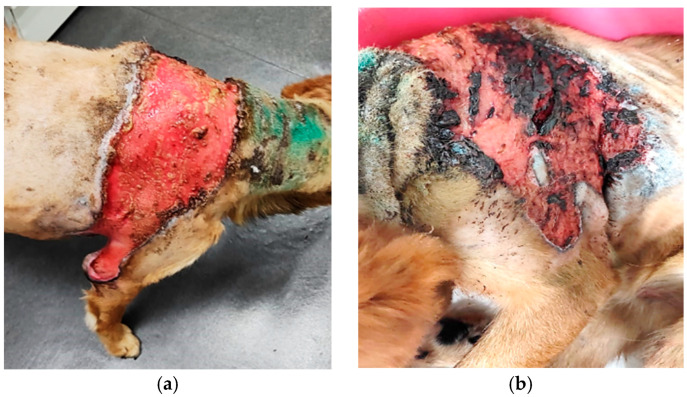
Toxic epidermal necrolysis: (**a**) severe and diffuse ulceration on the dorsum; (**b**) skin lesions on anterior limbs with gangrenous areas.

Case presentation: The patient was an 11 year-old, cross-breed intact male dog with an aggressive behaviour. History data suggested a previous injury caused by biting and fighting with another dog from the country habitat. The patient was found to be expressing clinical signs of shock, such as hypotension, weakness, depression, anorexia and fever concurrently with dermatological lesions. These consisted of severe and diffuse ulceration on the dorsum with symmetrical extension to the anterior limbs with gangrenous areas (Figure 1). Under supportive therapy with Duphalyte and enrofloxacin, the patient had become relatively recovered before our investigation.

Cytology: Skin samples were collected with sterile cotton swabs from different affected areas, and multiple smears were prepared and stained using May Grünwald-Giemsa and Gram stains. Microscopic examination identified intense neutrophil infiltration with the presence of free and phagocytized Gram-positive cocci.

Bacterial culture and antimicrobial susceptibility testing: Skin exudates were sampled using eSwabs^®^-Copan (Copan, Brescia, Italy) in Amies liquid. Samples were inoculated in duplicate on 5% sheep Blood and MacConkey Agar (HiMedia Laboratories GmbH, Einhausen, Germany) and incubated aerobically at 37 °C for 24–48 h. A significant mixed culture with haemolytic and non-haemolytic colonies of Gram-positive cocci was observed only on a Blood Agar (BA) plate.

The antimicrobial susceptibility of clinical isolates was determined by performing the Kirby–Bauer disk diffusion method using the protocol specified in the guidelines of the Clinical and Laboratory Standards Institute [CLSI]. The sensitivity pattern of the isolates was determined against 15 antibiotics: oxacillin, methicillin, cefalexin, cefuroxime, amoxyclave, spectinomycin, lincomycin, rifampicin, clarithromycin, doxycycline, oxytetracycline, trimethoprim-sulfamethoxazole, vancomycin, enrofloxacin and levofloxacin.

Species confirmation was performed using biochemical and molecular testing: catalase, mannitol fermentation, coagulase, latex agglutination, detection of the *nuc* thermonucleaze gene via real–time PCR methods for *S. aureus* identification [19] and Whole Genome Sequencing with metagenomic analysis for *S. halichoeri* identification. Additionally, the *S. aureus* strain was checked for 10 enterotoxin genes (*sea*, *seb*, *sec*, *sed*, *seg*, *seh*, *sei*, *sej*, *ser*, *sep*) using a multiplex real-time PCR method [19], but also for the genes causing methicillin resistance (*mecA*, *mecC*), protein A (*spa*) [20], toxic shock syndrome toxin-1 (*tst*) and exfoliative toxins (*eta*, *etb*) using a conventional PCR method [21,22,23]. Unfortunately, *Dermatophilus* spp. was presumed only morphologically in cytological preps and not through culturing, probably being inhibited by polymicrobial competition.

The therapeutic protocol began with shock therapy including Duphalyte and Enroxil, 10 mg/kg intravenously, every 24 h, for 5 days. Topically, Chlorhexidine solution 2% was used for cleaning the wound. We decided to interrupt the enrofloxacin administration after *S. canis* suspicion based on cytology because of the high risk of pathogen emergence caused by superantigen expression through the enrofloxacin-lysis of *S. canis* [14]. The treatment continued with clindamycin 10 mg/kg for 60 days with good clinical recovery. At 30 days after therapy discontinuation, clinical relapse occurred and the treatment was changed to the oral administration of trimethoprim sulfamethoxazole 17 mg/kg, for 60 days.

Unfortunately, we could not perform therapy monitoring regularly according to standard protocols (every 2–3 weeks), but only after clinical relapse, depending on the owner’s motivation. Finally, several cycles of antibiotic therapy were necessary to complete clinical recovery.

## 3. Results

History and clinical signs were consistent with toxic shock syndrome-like, including hypotension, weakness, depression, anorexia and fever concurrent with severe and diffuse ulcerative dermatitis on the dorsum with symmetrical extension to anterior limbs with gangrenous areas (Figure 1). Dermatological lesions led us to suspicions of thermal or chemical burn, drug reactions, epitheliotropic lymphoma or deep fungal infection.

**Figure 2:**
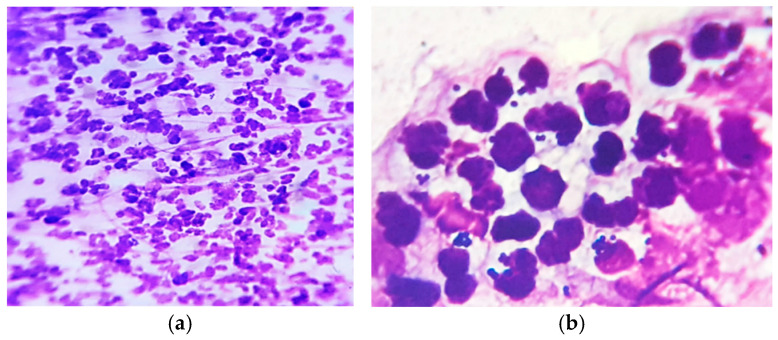
Skin cytology: (**a**) high cellular infiltrate with degenerate neutrophils (MGG stain, ×200); (**b**) some neutrophils with phagocytized cocci (MGG stain, ×1000).

Skin cytology from different affected areas was useful for diagnosis, showing intense neutrophil infiltration, with hyper segmented and degenerate neutrophils, some of them entrapped in a fibrin matrix, containing phagocytized cocci (Figure 2).

**Figure 3:**
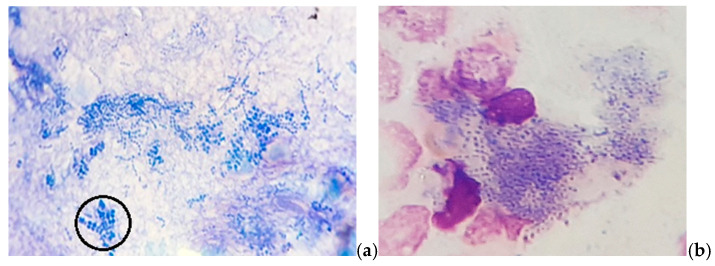
Skin cytology: (**a**) abundant free small coccoid cells with a “railroad track” appearance (in the circle); (**b**) phagocytized small coccoid cells of presumptive *Dermatophilus* spp. (MGG stain, ×1000).

**Figure 4:**
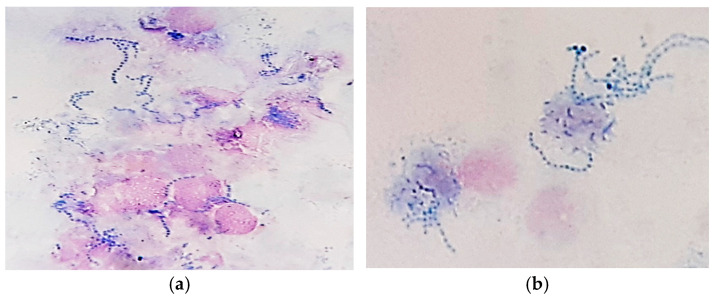
Skin cytology: (**a**) free and phagocytized streptococci (MGG stain, ×1000); (**b**) phagocytized streptococci—detailed image (MGG stain, ×1000).

In MGG-stained smears, we also detected abundant clusters and rows of small coccoid cells of presumptive *Dermatophilus* spp., occasionally with a “railroad track” appearance (Figure 3), as well as numerous cocci chains (Figure 4).

**Figure 5:**
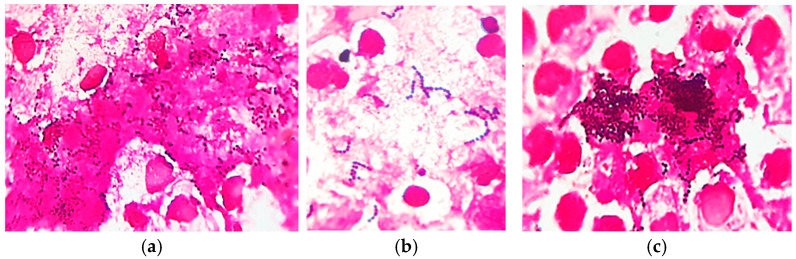
Skin cytology: (**a**) free and phagocytized Gram-positive cocci (Gram stain, ×1000); (**b**) short chains of streptococci (Gram stain, ×1000); (**c**) clusters of intense Gram-positive cocci (Gram stain, ×1000).

Gram-stained smears from skin lesions showed abundant free and phagocytized Gram-positive cocci, but only in a few examined fields (Figure 5).

**Figure 6:**
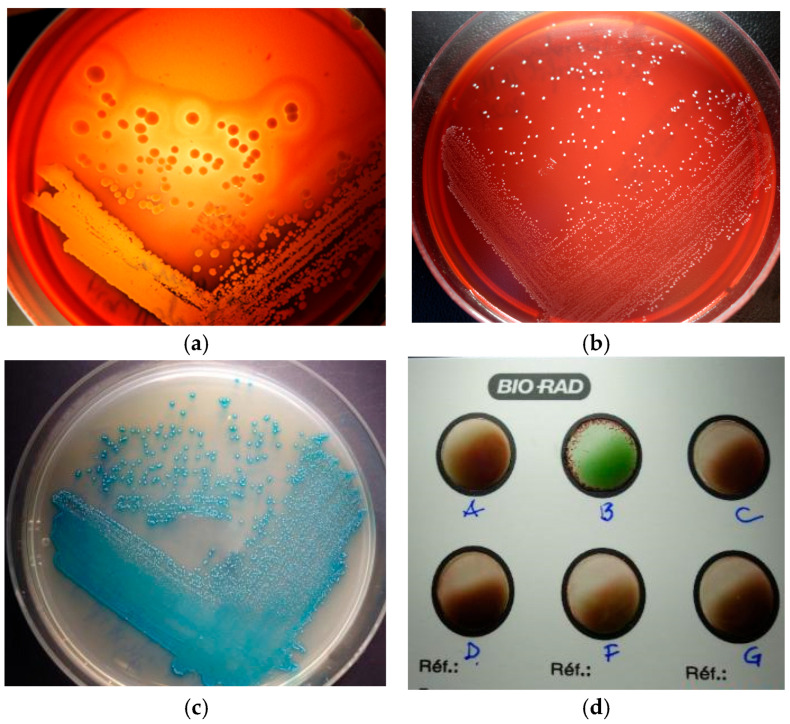
Bacterial culture: (**a**) double-haemolysis of *S. aureus* colonies; (**b**) non-haemolytic colonies of *S. halichoeri*; (**c**) βGRU-positive colonies of *S. halichoeri*; (**d**) *S. halichoeri*—positive agglutination with the Group B Lancefield.

Bacterial culture from skin exudates indicated, only on Blood Agar (BA) plate, a significant mixed culture with cream-yellow smooth, medium α-β haemolytic colonies and whitish-grey, pin-points umbonate, non-haemolytic colonies. Each colony type was picked-up for Gram’s staining and subcultured into Brain-Heart-Infusion (HiMedia Laboratories GmbH, Einhausen, Germany), Blood Agar (Figure 6a,b), Mannitol Salt Agar and Hichrome UTI-Agar (HiMedia Laboratories GmbH, Germany) for presumptive bacterial identification. Gram staining from agars showed intense Gram-positive cocci, in clusters and short chains, but substantial chaining was revealed when pin-point colonies were grown in BHI, an important feature for the streptococci group. On MSA-Agar, our staphylococcal isolate showed mannitol-positive golden yellow colonies. On the chromogenic UTI agar, our streptococci revealed βeta-glucuronidase (βGRU)-positive colonies with a turquoise pigment, resembling those of enterococci (Figure 6c) Additionally, our strain gave a positive agglutination reaction with the Group B Lancefield antisera (Pastorex Strep, Bio-Rad, Hercules, CA, USA)—Figure 6d. Finally, we considered our isolate to be of the non-haemolytic *Streptococcus* Group B. Taken together, the colony morphology, double-haemolysis, catalase, mannitol fermentation and coagulase-positive tests and strong agglutination with the Prolex Staph Latex Kit (Pro-Lab Diagnostics, Richmond Hill, ON, Canada) were significant for *S. aureus* identification.

Susceptibility testing showed the sensitivity of our *S. aureus* strain to lincosamides, sulphonamides, fluoroquinolones, rifampicin and vancomycin, but the resistance to all beta-lactam antibiotics and tetracyclines. The *Streptococcus* isolate was sensitive to all antimicrobials tested.

Molecular testing of the clinical isolate confirmed the diagnostic through the detection of the *nuc* thermonucleaze gene using the real-time PCR method (for *S. aureus* confirmation) and Whole Genome Sequencing with metagenomic analysis (for *S. halichoeri* confirmation). Additionally, PCR analyses of our *S. aureus* strain demonstrated the presence of the genes for enterotoxin H (*seh*), protein A (*spa*), toxic shock syndrome toxin TSST-1 (*tst*) and methicillin resistance (*mecC*), but the lack of genes for exfoliative toxins (*eta*, *etb*).

**Figure 7:**
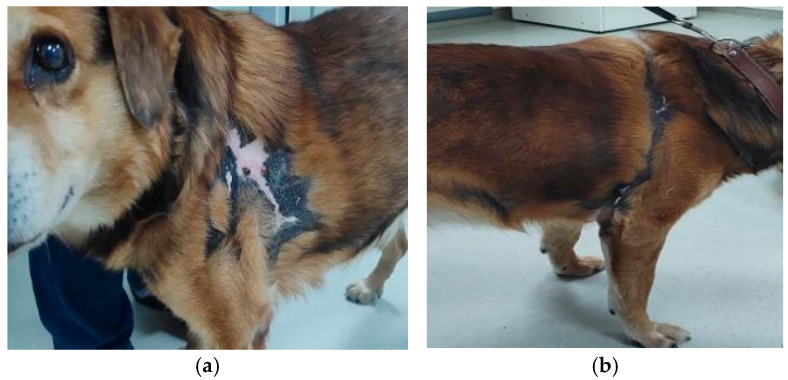
Complete clinical recovery, (**a**)—left and (**b**)—right side, after 1 year of therapy.

Therapeutic protocol: The patient fully recovered after several cycles of antibiotic therapy lasting for about one year, with periodical relapse (Figure 7).

**Figure 8:**
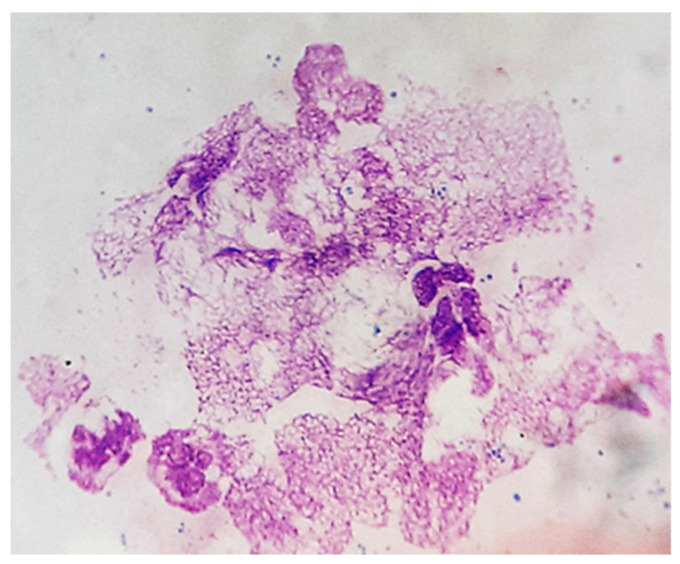
Skin cytology. Particular morphology of neutrophils with nuclear networks (MGG stain, ×1000).

Surprisingly, after two cycles of antibiotic therapy, we identified, via the cytological examination of chronic skin wounds, the presence of neutrophils with a particular morphology (Figure 8), which we assumed was suggestive of neutrophil extracellular traps (NETs). These are defined as extruded chromatin coated with antibacterial molecules and enzymes, which can kill microorganisms even when the neutrophils have died [2]. NETs are usually abundant at sites of infection, indicating the activation of antimicrobial innate immunity.

**Figure 9:**
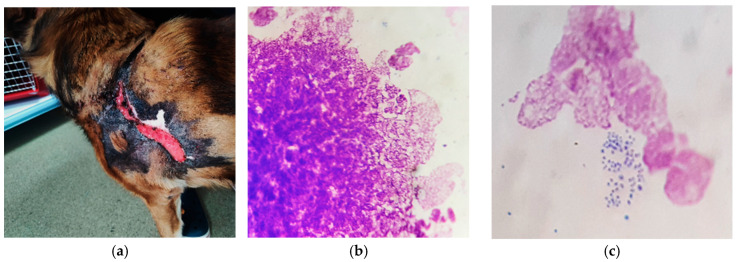
(**a**) Delayed wound healing; (**b**) excessive neutrophil nuclear networks (MGG stain, ×1000); (**c**) persistent bacterial colonization, after 6 months of therapy (MGG stain, ×1000).

However, excessive NET formation may amplify inflammation and tissue damage leading to delayed wound healing (Figure 9a–c) due to their toxic components, such as histones, myeloperoxidase, matrix metalloproteinases, etc. [24].

## 4. Discussion

In dogs, *S. aureus* and *Dermatophilus* spp. are not recognized as usual pathogens of the skin, but occasionally can cause severe ulcerative pyoderma leading to death. *S. halichoeri*, as a skin inhabitant in dogs, may be associated with abscesses and cellulitis, but is most frequently isolated in polymicrobial culture [14]. Particularly, *S. aureus* is known as the most versatile pathogen, able to readily cross species barriers and adapt to new hosts and niches via sophisticated attack and defence mechanisms, including toxin-mediated cytotoxicity, invasiveness, immune evasion and antibiotic resistance in correlation with host specific reactivity [2,3,5,6,7,9]. In our patient, we can speculate that *S. aureus* infection was transmitted from another carrier dog after biting trauma, while *Dermatophilus* spp. might have been secondarily acquired from the soil.

Both *S. aureus* and *S. pseudintermedius* are known to produce a number of potent superantigens, including staphylococcal enterotoxins (SEs), staphylococcal exfoliative toxin (ET) and toxic shock syndrome toxin (TSST-1), which are able to cause the release of massive amounts of host inflammatory cytokines, notably TNF-α. These molecules are responsible for the clinical and pathologic features of septic shock, including high fever, hypotension, haemoconcentration, thrombosis, neutrophil and endothelial activation, non-specific T-cell proliferation and multiple organ failure [2,5,12,13,14,24]. In our case, the immunological response profile associated with TSS was not assessed, and no cytokine profiling (e.g., TNF-α) or protein-level confirmation of toxin production was conducted. Other bacteria reported to be responsible for TSS and invasive infections in dogs, cats, domestic animals and humans are *Streptococcus canis* and *Streptococcus equi* subsp. *zooepidemicus*. Both streptococci are opportunistic pathogens often found as inhabitants of the skin and mucosal surfaces, with zoonotic potential. Additionally, human toxic shock caused by *S. pyogenes* resembles the canine TSS, and these streptococci are rarely transferable to dogs [25].

In the skin, staphylococcal superantigens can mediate epidermal necrolysis via multiple mechanisms, such as keratinocyte activation, vascular endothelial activation and innate and adaptive immunity activation (neutrophil, monocytes, infiltrating lymphocytes) [2,5,10]. Staphylococcal toxins can also be responsible for direct cytotoxicity toward keratinocytes, even in the absence of immune mechanisms [26]. Thus, significant differences have been shown in the action of staphylococcal toxins on human keratinocytes. For example, staphylococcal exfoliative toxin (ET) is able to cause the intercellular separation of granular-layer keratinocytes, independent of super antigenic properties. Alpha-toxin (α-haemolysin) was shown to induce more significant cytotoxic damage to human keratinocytes, in contrast to super antigenic toxins (TSST-1, ET, enterotoxins SEA, SEB) [10]. It is also reported that alpha-toxin plays an important role in gangrene generation by inducing ischemic coagulative necrosis in affected adjacent tissue [18], as it can be seen in our patient (Figure 1a,b). Additionally, alpha-toxin and staphylococcal protein A of *S. aureus* isolates from a patient with psoriasis and atopic dermatitis were found to be able to induce direct proinflammatory effects via TNF-α release from keratinocytes [10]. In other experimental studies, *S. aureus* enterotoxins and alpha-toxin were demonstrated to promote apoptosis in different cell types, like T cells, neutrophils, epithelial cells and endothelial cells [27].

The recruitment of neutrophils observed in cytological preps was shown to be critical for the clearing of *S. aureus* infection but also for the significant adhesion of *S. aureus* to keratinocytes and skin colonization, independent of staphylococcal virulence factors [2,9,12,28]. Besides its antimicrobial role, neutrophil overactivation in the initial stage of shock syndrome can be responsible for the induction of a compensatory anti-inflammatory response characterized by decreased macrophage activity and T-cell anergy with an increasing risk of secondary infections [13,29]. The survival of our patient after the initial stage of shock could be explained by this compensatory mechanism. Moreover, the detection of particular neutrophils (NETs) in the smears from chronic skin wounds could be interpreted as a mechanism of enhancing bactericidal activity and preventing bacterial dissemination [2,27,28,30]. On the other hand, skin inflammation associated with the release of NETs was shown to be significantly correlated with increased colonization, as well as the persistence of *S. aureus* in mouse skin [4,28,30]. Indeed, in our patient, poor wound healing with extensive hyperpigmentation, hair loss and focal vitiligo and scars was associated with increased neutrophil nuclear networks and remaining bacterial colonization after long-term antibiotic therapy (Figure 9a–c). Of note, the early diagnosis of TSS and prompt shock and multiple-antimicrobial therapy (penicillin G, clindamycin, lincomycin, erythromycin, potentiated sulfas, etc.) are essential for patient recovery, and still, there is always a questionable antibiotic therapy (Appendix A).

## 5. Conclusions

The correlation of the history, clinical signs, cytology, bacterial culture and response to systemic antibiotic therapy was suggestive of a TSS-like diagnosis with the major involvement of *S. aureus* and presumptive role of *Dermatophilus* spp. and *S. halichoeri*.

In this case, toxic shock symptoms, multidrug resistance and clinical relapse were correlated with the presence of the genes for enterotoxin H (*seh*), protein A (*spa*), toxic shock syndrome toxin TSST-1 (*tst*) and methicillin resistance (*mecC*), but a lack of genes for the exfoliative toxins responsible for staphylococcal scalded-skin syndrome (*eta, etb*) in our *S. aureus* clinical isolate. However, the detection of *tst* and *seh* genes is not, by itself, sufficient to support a TSS-syndrome. Staphylococcal enterotoxins, TSST-1 and protein A are recognized as having an important role in the pathogenesis of invasive staphylococcal infection, preventing a protective immune response and recurrent/persistent infection even with antibiotic and surgical therapy.

In polymicrobial infections, like in our case, the pathogenic role of each species remains uncertain due to potential microbial interactions and host factors that may influence disease expression.

## Data Availability

No new data were created.

## Appendix A. Challenge Question

What would be the clinical benefits of combined systemic Enroxil plus Clindamicin plus Trimetoprim sulphonamide in the treatment of canine toxic shock syndrome caused by an MRSA strain of *S. aureus*?

A. Good penetrability into deep pyoderma lesions and accelerated clinical resolution of infection.

B. Under-expression of bacterial superantigens (toxins) in the skin surface and reducing inflammation.

C. Preventing the selection of intracellular pathogenic variants of *S. aureus* more resistant to antibiotics and host defence and therefore the clinical management of relapses of skin microbiota and barrier functions, and therefore the prevention of blood stream infections.

D. All of the above.
